# The Role of Gut Microbiota in the Pathogenesis of Obesity and Food Addiction: The Importance of the Gut–Brain Axis and the Dopaminergic System

**DOI:** 10.3390/brainsci16060650

**Published:** 2026-06-19

**Authors:** Marta Żebrowska-Gamdzyk, Napoleon Waszkiewicz, Sylwia Chojnowska

**Affiliations:** 1 Faculty of Health Sciences, University of Lomza, Akademicka Street 14, 18-499 Lomza, Poland; martazebrowskag@gmail.com; 2 Department of Psychiatry, Medical University of Bialystok, pl. Wołodyjowskiego 2, 15-272 Bialystok, Poland; napwas@wp.pl

**Keywords:** microbiota, gut–brain axis, obesity, food addiction, dopaminergic system

## Abstract

Obesity is one of the most serious public health challenges worldwide and has reached the scale of a global epidemic. Its etiology is multifactorial and includes genetic, environmental, hormonal, and neurobiological factors. In recent years, increasing attention has been paid to the role of the gut microbiota in the regulation of energy metabolism, inflammatory processes, and the functioning of the gut–brain axis. An increasing body of evidence suggests that the gut microbiota may influence the dopaminergic system and eating behaviors through bacterial metabolites, immune pathways, and the vagus nerve. Disturbances in microbiota composition may contribute to the development of chronic low-grade inflammation and compulsive consumption of highly processed foods. This article discusses the concept of food addiction as a phenomenon involving loss of control over eating, excessive reward system reactivity, and dopaminergic dysfunction within the mesolimbic reward system. Particular attention is given to the role of the gut microbiota in modulating these processes, including the potential effects of selected commensal bacteria and the importance of dietary interventions such as the ketogenic diet in regulating the gut–brain axis. The presented data suggest that modulation of the gut microbiota may represent a promising supportive strategy in the treatment of obesity and disorders associated with compulsive eating. At the same time, it is emphasized that the current state of knowledge is largely preclinical and observational, highlighting the need for further translational and clinical studies.

## 1. Introduction

Obesity is currently one of the most serious public health challenges worldwide. According to the latest World Health Organization data, it has reached the scale of a global epidemic. In 2022, more than 890 million adults met the criteria for obesity, while approximately 2.5 billion adults worldwide were overweight or obese. Particularly alarming is the fact that the rapid increase in obesity prevalence affects all age groups. Obesity is not merely an aesthetic issue. Excess body weight significantly increases the risk of developing numerous chronic diseases, including type 2 diabetes, hypertension, cardiovascular diseases, non-alcoholic fatty liver disease, obstructive sleep apnea, and certain cancers [1,2]. Consequently, obesity leads not only to a deterioration in patients’ quality of life but also constitutes a substantial burden on healthcare systems worldwide. Currently, obesity is considered to have a multifactorial etiology that includes not only unhealthy dietary habits and low levels of physical activity but also genetic, environmental, hormonal, and neurobiological factors [1,3,4,5]. However, in recent years, increasing attention has been paid to the role of the gut microbiota in the pathogenesis of obesity and metabolic disorders. Numerous studies indicate that the composition and activity of microorganisms inhabiting the gastrointestinal tract may strongly influence the body’s energy metabolism, the initiation and maintenance of inflammatory processes, appetite regulation, and the functioning of the gut–brain axis (GBA) [6,7]. Growing evidence also suggests that the gut microbiota may affect the reward system and modulate eating behaviors through its influence on neurotransmitters, including dopamine [8]. Therefore, disturbances in the balance of the gut microbiota observed in individuals with obesity may contribute both to the development of metabolic disorders and to behavioral changes associated with excessive consumption of highly processed foods rich in sugars and trans fats [9]. The hedonic properties of food, such as taste, smell, and texture, may lead to overstimulation of dopaminergic pathways responsible for the sensation of pleasure and the motivation for excessive food intake [10,11].

Despite the growing number of therapeutic approaches, the treatment of obesity and disorders associated with compulsive food consumption remains a significant clinical challenge. The effectiveness of conventional interventions based solely on caloric restriction and increased physical activity is often limited, and the greatest difficulty lies in maintaining long-term therapeutic outcomes [6]. In turn, bariatric surgery and pharmacotherapy, although effective particularly in the treatment of complicated obesity [12], do not teach patients appropriate eating behaviors. As a result, unfavorable dietary habits may persist, contributing to the continued development of inflammation and obesity-related comorbidities despite weight reduction. These observations highlight the need to search for new therapeutic strategies that take into account the neurobiological mechanisms regulating eating behaviors. Increasing attention is being paid to the possibility of modulating the gut–brain axis through influencing the gut microbiota. Research findings suggest that intestinal microorganisms may affect the dopaminergic system through their metabolites, as well as via immune and hormonal pathways and direct signaling through the vagus nerve, thereby influencing mechanisms of reward, motivation, and appetite control [13].

If disturbances in the gut microbiota promote excessive consumption of highly processed foods rich in sugars and trans fats, and such a diet further exacerbates intestinal dysbiosis, this may lead to a self-perpetuating cycle of mutually reinforcing metabolic and behavioral disturbances resembling mechanisms observed in substance use disorders [10,14,15,16,17,18,19,20]. Therefore, microbiota-targeted therapeutic strategies may represent a promising supportive approach in the treatment of obesity [13].

## 2. Food Addiction

Food addiction is defined as the occurrence of intense, difficult-to-control cravings and a loss of control over eating behaviors, leading to compulsive consumption, particularly of palatable, highly processed foods [12]. Epidemiological data indicate that symptoms of food addiction affect approximately 14% of the adult population, with prevalence increasing to around 28% among individuals with obesity [21]. In adolescent populations, these rates are approximately 15% and 19%, respectively [22]. It should be emphasized that although symptoms of addiction to ultra-processed food are more common among individuals with obesity, obesity and food addiction are distinct but overlapping conditions [23]. In adults, the prevalence of food addiction is comparable to that of addictions to legal psychoactive substances such as alcohol and tobacco [24]. Although food addiction is not currently classified as a distinct disorder in either the International Classification of Diseases or Diagnostic and Statistical Manual of Mental Disorders, a growing body of evidence points to substantial similarities with substance use disorders [20]. Neuroimaging and preclinical studies demonstrate that ultra-processed foods rich in simple sugars, trans fats, and flavor-enhancing additives activate the mesolimbic reward system, including the ventral tegmental area (VTA) and nucleus accumbens (NAc), in a manner similar to addictive substances [24]. These processes involve disturbances in dopamine release dynamics and reduced availability of dopamine D2 receptors in the striatum, which represents one of the key mechanisms underlying substance addiction [25,26].

Additionally, food addiction is characterized by behavioral components typical of chemical addictions, such as loss of control, craving, and withdrawal symptoms [20,23]. However, phenomena such as cue reactivity, reward system sensitization, and impaired inhibitory control are also observed in food addiction, promoting the persistence of compulsive eating patterns. Similar mechanisms are present in behavioral addictions such as pathological gambling and compulsive shopping. These disorders do not require the intake of a specific substance but are instead based on repetitive behaviors that activate the reward system through mechanisms of positive reinforcement. Consequently, food addiction is increasingly conceptualized as a substance-like behavioral disorder that combines behavioral components with disturbances in the neurobiological mechanisms of reward [27,28,29].

Dopamine is a key neurotransmitter modulating reward processing, motivation, and associative learning, and the dopaminergic system constitutes a central element in the neurobiology of addiction. In the context of eating behaviors, increased dopaminergic activity plays an important role both in reward encoding and reward anticipation, thereby shaping the motivation to seek rewarding stimuli [30]. Regulation of dopaminergic function in response to rewarding stimuli occurs primarily through mesolimbic and mesocortical pathways. Dysregulation of these systems leads to impaired cognitive control, including decision-making, response inhibition, and evaluation of behavioral consequences. This process is associated with functional changes in the prefrontal cortex and basal ganglia structures, resulting in disruption of the balance between the reward system and executive control [31]. In the classical addiction model, psychoactive substances increase extracellular dopamine concentrations, among others, by inhibiting dopamine reuptake (e.g., cocaine), thereby enhancing rewarding effects and reinforcing compulsive behaviors [32]. Concomitantly, neuroadaptive changes in dopaminergic signaling have been observed, particularly involving reduced striatal D2/D3 receptor availability in individuals with substance use disorders, which may contribute to impaired reward processing and compulsive drug-seeking behaviors [33,34]. Similar phenomena have been described in experimental animal models exhibiting compulsive sugar consumption, in which decreased D2 receptor activity in the nucleus accumbens was also observed [35].

The Yale Food Addiction Scale (YFAS) is the most commonly used instrument for assessing the severity of food addiction. Its current version was developed based on the diagnostic criteria for substance use disorders. A diagnosis of food addiction is established when at least two out of eleven criteria related to the consumption of ultra-processed foods are fulfilled, in combination with clinically significant distress or impairment in functioning [36].

The development of addictions, including food addiction, is most commonly understood as the result of interactions among three groups of factors: the addictive properties of a given substance or stimulus, individual biological and psychological susceptibility (including genetic predisposition, mood disorders, or family history of addiction), and environmental factors that determine the availability, attractiveness, and exposure to the addictive stimulus [20]. The topic of hedonic eating is highly relevant to psychiatry because neurochemical changes in brain regions associated with pleasure and reward may respond well to psychiatric interventions currently used in the treatment of other addictions or even eating disorders [20,22].

## 3. The Role of the Gut Microbiota in Food Addiction

A growing body of evidence suggests that the gut microbiota may play a significant role in modulating the dopaminergic system through the gut–brain axis. Although dopamine synthesized in the gastrointestinal tract does not cross the blood–brain barrier, intestinal microorganisms may influence the neuroavailability of its precursors, such as tyrosine and L-DOPA, as well as modulate the activity of dopaminergic neurons through neuronal, immunological, and metabolic pathways [8,37]. It has been demonstrated that the microbiota affects the functioning of the mesolimbic reward system, including dopaminergic projections from the ventral tegmental area to the nucleus accumbens, which is responsible for processing rewarding stimuli, motivation, and reinforcement mechanisms [13].

A key component of bidirectional communication within the gut–brain axis is the vagus nerve, which connects the gastrointestinal tract with the central nervous system. Approximately 80% of vagal nerve fibers are afferent, meaning that they transmit information to the brain regarding intestinal status, nutrient composition, the presence of bacterial metabolites, and immune signals [38]. Afferent signals originating from the gut may modulate the activity of dopaminergic neurons, thereby influencing reward perception, motivation, and reinforcement-based learning mechanisms [13]. In this way, the vagus nerve constitutes a direct “neuronal connection” between the gut microbiota and the reward system. Disturbances in gut microbiota composition may therefore alter the reactivity of the dopaminergic system by modulating responses to highly processed food-related stimuli and promoting the persistence of compulsive eating patterns characteristic of food addiction. Furthermore, it has been suggested that chronic dysbiosis combined with prolonged consumption of foods rich in refined sugars and saturated trans fats may lead to neuroadaptations within the reward system, including alterations in dopaminergic neurotransmission and regulation of dopamine D2 receptors [13]. These mechanisms may represent one of the biological links connecting gut microbiota disturbances, metabolic disorders, and the development of compulsive eating behaviors. Increasing evidence indicates that the influence of the microbiota on the dopaminergic system extends beyond neurotransmitter metabolism and also includes modulation of neuroinflammation, intestinal barrier integrity, vagus nerve activity, and the production of bacterial metabolites involved in neuronal signaling [13,37].

The main bacterial taxa potentially involved in gut–brain communication and dopaminergic modulation are summarized in Table 1.

Animal studies have demonstrated that certain strains of *Lactobacillus*, particularly *Lactobacillus rhamnosus* and *Lactobacillus plantarum*, may influence central nervous system activity through modulation of the vagus nerve, regulation of neurotransmitter receptor expression, and reduction in the stress response [39]. *Lactobacillus rhamnosus* has been shown to alter the expression of GABA receptors in the mouse brain and to reduce anxiety- and depressive-like behaviors by attenuating excessive activation of the stress axis and modulating neurotransmitter activity [40]. Importantly, these effects disappear following vagotomy, suggesting a crucial role of the gut–brain axis in this mechanism.

*Lactobacillus plantarum* demonstrates even stronger associations with the dopaminergic system. The best-described psychobiotic strain is *Lactobacillus plantarum PS128*. Animal studies have shown that *PS128* modulates dopamine and serotonin levels, reduces anxiety- and depressive-like behaviors, influences mesocortical and nigrostriatal pathways, and affects the functioning of the microbiota–gut–brain axis. It has also been demonstrated that *PS128* alleviates hyperactive tic-like behaviors through stabilization of cerebral dopaminergic pathways mediated by the microbiota–gut–brain axis. Consequently, there is considerable potential for the use of *Lactobacillus species* as psychobiotics aimed at improving symptoms associated with dopaminergic dysregulation. However, these findings are derived predominantly from animal models, and their clinical relevance in obesity and food addiction remains to be established [41,42].

Among the best-studied gut microbiota microorganisms in the context of the gut–brain axis are bacteria of the genus *Bifidobacterium*. Increasing evidence indicates that these bacteria may influence nervous system function, stress-response regulation, neuroinflammation, and mechanisms associated with reward and addictive behaviors. Although the direct influence of *Bifidobacterium* on dopaminergic transmission remains under investigation, numerous findings suggest their indirect involvement in modulation of the dopaminergic system. Specific *Bifidobacterium* strains have been shown to affect tryptophan metabolism and regulate the production of short-chain fatty acids (SCFAs), which influence gut–brain axis functioning [37,43].

Studies concerning obesity and compulsive eating have shown that reduced abundance of *Bifidobacterium* coexists with chronic inflammation, increased intestinal permeability, metabolic disturbances, and intensified behaviors related to excessive consumption of highly processed foods [13,37,44,45]. Some studies also suggest that restoration of *Bifidobacterium* populations may improve communication within the gut–brain axis and reduce reward system hyperreactivity [44].

In the context of catecholamine metabolism, bacteria of the genus *Enterococcus* and the species *Escherichia coli* are also of particular interest. Certain strains of these microorganisms are capable of synthesizing or metabolizing compounds that serve as neurotransmitter precursors, including tyrosine and L-DOPA [8].

In recent years, increasing attention has been devoted to *Akkermansia muciniphila*, a bacterial species associated with proper intestinal barrier integrity and regulation of metabolic and inflammatory processes. Research findings indicate that reduced abundance of *Akkermansia muciniphila* may coexist with obesity, insulin resistance, and chronic low-grade inflammation [46]. It is also increasingly suggested that, through its influence on intestinal permeability and neuroinflammation, this bacterium may indirectly modulate dopaminergic system functioning and improve communication within the gut–brain axis.

One of the most interesting aspects of research on *Akkermansia muciniphila* is its association with eating behaviors. Numerous studies have shown that individuals with compulsive eating behaviors and metabolic disturbances exhibit reduced levels of this bacterium [47]. It is increasingly proposed that chronic dysbiosis involving reduced abundance of *Akkermansia muciniphila* may contribute to persistent disturbances in the gut–brain axis and excessive activation of the reward system by highly processed foods [46].

Particularly noteworthy are recent reports concerning *Blautia wexlerae*, whose reduced abundance has been observed in individuals exhibiting features of food addiction [48]. *Blautia wexlerae* belongs to the commensal gut microbiota bacteria currently being investigated in the context of obesity, metabolic disorders, and the gut–brain axis. Although studies examining its influence on the dopaminergic system and addiction remain limited, increasing evidence suggests that it may indirectly affect mechanisms involved in appetite regulation, inflammation, and eating behaviors. However, unlike psychobiotics such as *Lactobacillus* or *Bifidobacterium*, the effects of *Blautia wexlerae* appear to be primarily metabolic and immunomodulatory in nature [49].

Most available evidence concerning psychobiotics in the context of food addiction and the dopaminergic system remains preclinical. Therefore, it is still not possible to discuss the treatment of addiction using probiotics. Moreover, current knowledge indicates that the influence of the gut microbiota on the dopaminergic system is multifactorial and does not depend on the action of a single bacterial strain. Interactions among bacterial metabolites, the immune system, the vagus nerve, and neurotransmitter regulation within the gut–brain axis appear to play a crucial role. These mechanisms may represent one of the biological foundations of impaired appetite control, compulsive consumption of highly processed foods, and the development of food addiction. Consequently, dietary strategies aimed at supporting bacterial strains considered beneficial for regulation of the dopaminergic system are of particular importance.

## 4. Dopamine, Gut Microbiota, and Diet

It has been suggested that the ketogenic diet may have a particularly beneficial effect on the dopaminergic system. It has been demonstrated that the use of a ketogenic diet in adult male mice reduces their preference for alcohol consumption [50] and attenuates behavioral responses to cocaine in rats [51]. Several scientific studies have also described the benefits of the ketogenic diet in individuals with food addiction [52,53]. It is also noteworthy that the ketogenic diet may alter the composition of cell membrane lipids, thereby influencing neurotransmitter release and membrane receptor function [54].

An increasing number of studies also indicate that the ketogenic diet can significantly affect the composition and activity of the gut microbiota and thus indirectly modulate the functioning of the GBA and the dopaminergic system [47,55]. These findings appear particularly relevant in the context of metabolic disorders, obesity, and food addiction. The ketogenic diet is a nutritional model based on a substantial reduction in carbohydrate intake and an increased proportion of fats, leading to the induction of ketosis and the use of ketone bodies as an alternative energy source [56].

However, it is currently emphasized that the metabolic effects of the ketogenic diet may depend on the quality of dietary fats and the degree of processing of consumed foods. A so-called “well-formulated ketogenic diet” is based mainly on natural, unrefined fat sources, vegetables, and nutrient-dense foods [55,57], whereas a ketogenic diet based on ultra-processed foods, trans fats, and refined or hydrogenated vegetable oils may promote inflammatory processes and unfavorable changes in the gut microbiota [47,58]. Specifically, such products may lead to a reduction in beneficial bacterial populations, including *Bifidobacterium* and *Akkermansia muciniphila*, while increasing bacteria associated with inflammation and metabolic endotoxemia [59]. Recent experimental evidence has provided additional insight into the cellular mechanisms linking obesogenic dietary patterns with intestinal dysfunction. Using human small-intestinal organoid models, Filippello et al. demonstrated that chronic exposure to palmitate, one of the predominant saturated fatty acids present in ultra-processed foods, impairs the differentiation of enterocytes, goblet cells, and cholecystokinin-secreting enteroendocrine I cells. Importantly, palmitate exposure reduced MUC2 expression and mucin secretion, indicating direct impairment of goblet cell function and mucus barrier homeostasis. These findings suggest that saturated fatty acids may contribute to gut microbial dysregulation not only through dietary effects on microbiota composition but also through direct adverse effects on intestinal epithelial differentiation and mucus barrier maintenance. Such mechanisms may represent an important link between ultra-processed food consumption, dysbiosis, and gut–brain axis dysfunction associated with obesity and maladaptive eating behaviors [60]. Chronic exposure to highly processed fats may therefore impair intestinal barrier integrity, activate immune responses, and promote neuroinflammation, all of which are considered mechanisms that disrupt the functioning of the mesolimbic reward system.

Only a “well-formulated diet” may contribute to the restoration of beneficial bacterial populations and be associated with improved metabolic parameters and appetite regulation [55,57]. These mechanisms may include reduced hedonic stimulation induced by refined sugars and decreased chronic activation of the mesolimbic reward system. One of the most frequently observed effects of a well-designed ketogenic diet is a significant increase in *Akkermansia muciniphila*. As previously mentioned, this bacterium is associated with improved intestinal barrier integrity, reduced metabolic endotoxemia, and decreased chronic low-grade inflammation characteristic of obesity [47].

Particular interest has been directed toward the impact of natural saturated fats in the ketogenic diet, especially medium-chain triglycerides (MCTs) found in coconut oil. Experimental studies have shown that diets rich in MCTs may promote the growth of *Akkermansia muciniphila* and selected *Lactobacillus strains* [61]. This mechanism is thought to be related to the distinct metabolism of MCTs, which undergo faster β-oxidation and induce less metabolic endotoxemia than long-chain saturated fats.

It has also been shown that the Mediterranean diet, rich in unrefined plant-based fats, is associated with increased production of short-chain fatty acids, which may influence appetite regulation and gut–brain axis functioning. Unrefined vegetable oils such as extra virgin olive oil, flaxseed oil, and avocado oil, rich in monounsaturated and polyunsaturated fatty acids, also contain polyphenols and antioxidant compounds that may support the growth of *Bifidobacterium* and *Lactobacillus* while limiting the expansion of pro-inflammatory bacteria [62].

Although some studies report a transient reduction in *Bifidobacterium* abundance during a restrictive ketogenic diet, it has been hypothesized that this effect may result from a sudden reduction in fermentable carbohydrates, with levels potentially recovering over time [60]. Nevertheless, findings regarding the effects of ketogenic diets on gut microbiota remain heterogeneous. While several studies report beneficial increases in taxa such as *Akkermansia muciniphila*, others have observed reductions in microbial diversity or decreases in bacterial groups generally considered beneficial, including *Bifidobacterium* spp. These discrepancies may result from differences in study duration, dietary composition, participant characteristics, and the degree of carbohydrate restriction. Therefore, the long-term effects of ketogenic diets on gut microbiota composition and gut–brain axis function remain incompletely understood and require further investigation.

Experimental models have also shown that the ketogenic diet may influence vagus nerve activity, GABAergic transmission, and dopaminergic system function, potentially modulating reward-related behaviors and impulsivity [61,63]. The ketogenic diet also appears to support the metabolism of neurotransmitter precursors and the production of short-chain fatty acids. These compounds act on enteroendocrine cells, modulate immune responses, and influence gut–brain axis activity, potentially affecting dopaminergic projections between the ventral tegmental area and the nucleus accumbens.

It has been suggested that by reducing chronic exposure to highly rewarding ultra-processed foods, a well-formulated ketogenic diet may partially normalize excessive activation of the reward system and reduce compulsive eating behaviors. Thus, the effectiveness of a properly balanced ketogenic diet may stem from both metabolic changes and potential neurobiological effects related to reward, impulsivity, and hedonic food regulation. This makes the gut–brain–microbiota axis one of the most promising directions in research on food addiction.

**Figure 1:**
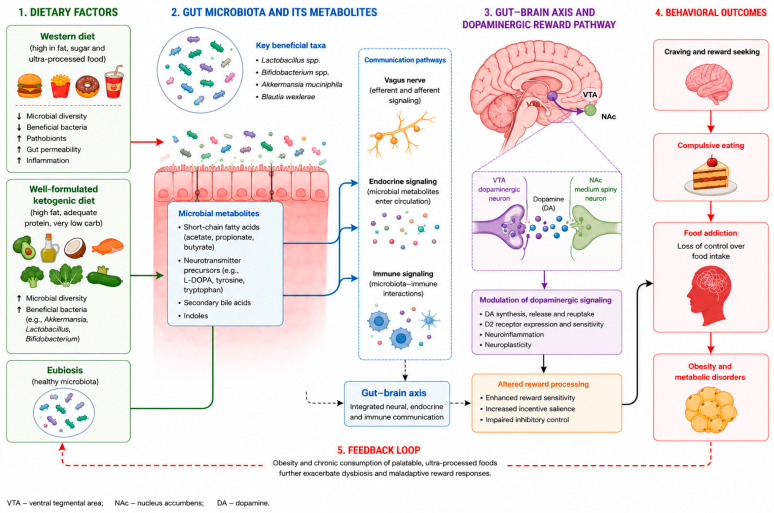
Potential mechanisms linking diet, gut microbiota, dopaminergic signaling, food addiction, and obesity through the gut–brain axis.

The complex interactions among diet, gut microbiota, gut–brain communication, dopaminergic reward pathways, food addiction, and obesity are summarized in Figure 1.

From a therapeutic perspective, modulation of the gut microbiota should currently be regarded as a promising but still developing supportive strategy rather than an established treatment for food addiction. Meta-analyses of randomized controlled trials suggest that probiotic supplementation may produce modest improvements in obesity-related outcomes, including body weight, BMI, and body fat percentage; however, the observed effects are generally small and characterized by considerable heterogeneity among studies, likely reflecting differences in bacterial strains, dosage, intervention duration, baseline microbiota composition, and concomitant lifestyle modifications [64].

Psychobiotics represent another emerging therapeutic approach. Although preclinical studies indicate that selected bacterial strains may influence stress responses, neurotransmitter-related pathways, and gut–brain communication, clinical evidence directly linking psychobiotic interventions to improvements in food addiction symptoms or dopaminergic reward dysfunction remains limited [65,66].

Similarly, dietary interventions, including well-formulated ketogenic diets, have been proposed as potential supportive strategies for individuals with obesity, binge eating, and food addiction symptoms. Preliminary clinical observations and pilot studies have reported reductions in food cravings, binge eating episodes, and Yale Food Addiction Scale scores following ketogenic dietary interventions. However, the available evidence is based primarily on case series and small pilot studies rather than adequately powered randomized controlled trials, limiting the strength of current conclusions [52,53].

Several important barriers to clinical translation should also be acknowledged. These include the absence of standardized therapeutic protocols, substantial interindividual variability in microbiota composition, strain-specific effects of probiotics and psychobiotics, and the limited use of food addiction-specific outcomes, such as validated food addiction scales, neuroimaging biomarkers, or measures of reward system activity. Consequently, microbiota-targeted interventions should currently be considered a promising area of translational research rather than an evidence-based therapeutic standard.

## 5. Strengths and Limitations of Current Evidence

The growing body of research investigating the interactions between gut microbiota, the gut–brain axis, and dopaminergic signaling has substantially expanded our understanding of the mechanisms potentially involved in the development of obesity and food addiction. One of the major strengths of the current literature is the increasing consistency of findings linking gut dysbiosis with metabolic disturbances, chronic low-grade inflammation, and alterations in reward-related behaviors. Advances in microbiome research have also enabled the identification of specific microbial taxa, including *Lactobacillus* spp., *Bifidobacterium* spp., *Akkermansia muciniphila*, and *Blautia wexlerae*, which may participate in gut–brain communication and indirectly influence dopaminergic function [13,37].

Another important strength of contemporary research is the growing understanding of the multidirectional communication pathways within the gut–brain axis. Evidence indicates that the effects of gut microbiota on central nervous system function may be mediated not only by microbial metabolites but also through immune signaling pathways, regulation of intestinal barrier integrity, neuroinflammatory processes, and vagus nerve activity [37,38]. This multidimensional perspective provides a more comprehensive understanding of the biological links between metabolic disturbances and compulsive eating behaviors.

Despite these advances, several important limitations remain. First, most available evidence is derived from preclinical animal studies, whereas the number of well-designed interventional studies in humans remains limited. Although experimental studies provide valuable insights into potential biological mechanisms, their findings cannot be directly extrapolated to human populations [13]. Second, substantial heterogeneity exists among studies with respect to microbiota assessment methods, dietary interventions, participant characteristics, and outcome measures, making direct comparisons difficult and limiting the ability to draw definitive conclusions [67].

Another major limitation is the difficulty in establishing causal relationships between alterations in gut microbiota composition and behaviors associated with food addiction. Most available studies are observational in nature and therefore cannot determine whether microbiota disturbances contribute to the development of obesity, maladaptive dietary habits, and compulsive eating or whether they arise as a consequence of these conditions [13]. Furthermore, accumulating evidence suggests that the effects of probiotics and psychobiotics are highly strain-specific, which complicates the generalization of findings across broader bacterial genera or microbial groups [65,66].

It should also be emphasized that despite growing interest in food addiction as a potential contributor to obesity, the concept remains a subject of scientific debate and has not been formally recognized as a distinct diagnostic entity in current psychiatric classification systems. This issue should be taken into account when interpreting existing findings and highlights the need for further research integrating microbiological, neurobiological, behavioral, and clinical perspectives [27].

Overall, current evidence suggests that gut microbiota may play an important role in modulating reward-related pathways involved in obesity and food addiction. However, a more complete understanding of these relationships requires further translational research and well-designed clinical studies capable of evaluating the therapeutic potential of microbiota-targeted interventions in the management of obesity and disordered eating behaviors.

## 6. Future Directions

Despite the growing body of evidence linking the gut microbiota, dopaminergic system function, and compulsive eating behaviors, most available studies remain observational and correlational in nature. This means that one of the most important challenges is to move from identifying co-occurring phenomena toward determining true cause-and-effect relationships and the underlying biological mechanisms.

Future research should place particular emphasis on studies that integrate experimental data with clinical observations to determine whether modulation of the gut microbiota can influence mesolimbic reward system activity and eating behaviors. Such studies should investigate microbiota-targeted approaches, including dietary and microbial interventions, to determine their effects on reward system activity and eating behaviors.

Another key direction is the identification not only of specific bacterial strains but also of their metabolites and associated signaling pathways involved in gut–brain communication. A better understanding of the mechanisms linking gut dysbiosis with reward system function may facilitate the identification of new therapeutic targets for the treatment of obesity and disorders associated with compulsive consumption of highly processed foods.

Future research should also consider the impact of different dietary patterns on gut microbiota composition, inflammatory processes, and dopaminergic system activity. Understanding these relationships may help explain individual susceptibility to maladaptive eating patterns and variability in response to therapeutic interventions.

Future therapeutic strategies for food addiction may require a multidimensional approach, simultaneously targeting the gut microbiota, neuroinflammatory processes, and reward regulation mechanisms, including interventions that activate the vagus nerve.

## 7. Conclusions

Obesity is a disease with a complex, multifactorial etiology, in which increasing importance is attributed to interactions between the gut microbiota, the immune system, and the central nervous system. A growing body of evidence indicates that dysbiosis may influence the functioning of the gut–brain axis and modulate dopaminergic system activity, which may be relevant for the regulation of appetite and eating behaviors.

The concept of food addiction emphasizes the role of neurobiological mechanisms, particularly the reward system and dopamine signaling, in the development of compulsive eating. These mechanisms show significant similarities to both substance-related and behavioral addictions, including loss of control, craving, and altered reward system responsiveness.

The gut microbiota may represent an important link between metabolic and neurobehavioral factors, influencing inflammation, intestinal barrier integrity, and the production of metabolites that affect the nervous system. In this context, microbiota-targeted and gut–brain axis-oriented interventions may represent a promising therapeutic direction.

At the same time, the current state of knowledge is mainly preclinical and observational in nature; therefore, further studies are necessary to determine the causal role of the microbiota in the development of food addiction and its potential clinical significance in the treatment of obesity.

## Figures and Tables

**Table 1:** Key microbiota-related factors and pathways implicated in dopaminergic signaling and reward regulation relevant to food addiction.

Microbiota-Related Taxon or Pathway	Proposed Mechanism	Potential Relevance to Dopamine/Reward Pathways	Current Evidence Base
*Lactobacillus rhamnosus*	Vagus nerve-dependent signaling; modulation of GABA receptor expression; regulation of stress responses	May indirectly influence reward-related behaviors through modulation of stress, anxiety, and emotional regulation	Primarily animal studies
*Lactobacillus plantarum PS128*	Modulation of gut–brain communication and dopamine- and serotonin-related pathways	May influence dopamine-related signaling and behavioral domains relevant to mood, anxiety, and impulsivity	Animal studies with limited human evidence
*Bifidobacterium* spp.	SCFA production; tryptophan metabolism; gut–brain communication	May indirectly affect reward-related pathways through microbial metabolites and neurotransmitter precursor metabolism	Animal and human studies
*Akkermansia muciniphila*	Improvement of intestinal barrier function; reduction in metabolic endotoxemia and neuroinflammation	May indirectly support gut–brain axis function and dopaminergic signaling through improved metabolic and inflammatory status	Animal and human studies
*Blautia wexlerae*	SCFA-related metabolic effects and modulation of inflammatory processes	May be associated with reduced susceptibility to food addiction and improved metabolic regulation; direct effects on dopaminergic signaling remain unclear	Emerging preclinical and observational human evidence
Vagus nerve (gut–brain pathway)	Bidirectional neural communication between the gut and the central nervous system	May contribute to modulation of mesolimbic dopamine activity and reward-related behaviors	Animal, clinical, and mechanistic evidence

## Data Availability

The original contributions presented in this study are included in the article. Further inquiries can be directed to the corresponding author.

## Abbreviations

The following abbreviations are used in this manuscript:

GBA	Gut–brain axis
MCTs	Medium-chain triglycerides
NAc	Nucleus accumbens
SCFAs	Short-chain fatty acids
VTA	Ventral tegmental area
